# Editorial: Basic, clinical, and translational studies of Yao syndrome and other NOD2 related diseases

**DOI:** 10.3389/fimmu.2024.1521815

**Published:** 2024-11-15

**Authors:** John M. Davis, Christine McDonald, Qingping Yao

**Affiliations:** ^1^ Division of Rheumatology, Mayo Clinic, Rochester, MN, United States; ^2^ Department of Inflammation and Immunity, Lerner Research Institute, Cleveland Clinic, Cleveland, OH, United States; ^3^ Division of Rheumatology, Allergy and Immunology, Stony Brook University Renaissance School of Medicine, Stony Brook, NY, United States

**Keywords:** autoinflammatory disease, inflammatory disease, NLR, NOD2, periodic fever syndrome, genetic variant, Yao syndrome, Blau syndrome

Nucleotide-binding oligomerization domain containing protein 2 (NOD2) is a member of NOD-like receptor family (NLRs) (1). The NLR family was first discovered in the early 2000s through the complementary efforts of several research teams, which resulted in multiple names for the same molecule (2–6). The family nomenclature has since been standardized; however earlier literature may refer to NOD2 as caspase recruitment domain-containing protein 15 (CARD15) based on its molecular structure that includes two N-terminal CARDs. NOD2 is an intracellular microbe sensor that plays important roles in infection defense, control of inflammation, and apoptosis (7). The discovery that *NOD2* missense and frameshift mutations are risk factors for the inflammatory bowel disease Crohn’s disease demonstrates the importance of this molecule in inflammatory disease (8, 9). Additionally, genetic variants of *NOD2* are linked to other autoinflammatory diseases, such as Blau syndrome and Yao syndrome (10); however, much more needs to be done to refine clinical phenotypes of NOD2-associated diseases and understand the molecular mechanisms underlying disease pathogenesis. The Research Topic “*Basic, clinical, and translational studies of Yao syndrome and other NOD2 related diseases*” brings together several articles to provide further insights into this area and proposes a new concept for defining autoinflammatory diseases.

In 2002, several highly penetrant variants on exon 4 of *NOD2* were found to cause Blau syndrome (BS, OMIM#186580), a pediatric (early onset) autosomal dominant granulomatous disease characterized by granulomatous arthritis, uveitis, and dermatitis (11). Initially, these *NOD2* variants were thought to result in overactive NOD2-dependent pro-inflammatory signaling (12); however, more recent studies indicate that the loss of NOD2 cross-regulatory function controlling the activity of other inflammatory pathways (13) may be a greater contributor to the pathobiology of BS. In this Research Topic, two articles investigate strategies targeting these altered NOD2 activities in novel treatment approaches for BS. Multiple immune suppressive therapies have been empirically employed as BS treatments (14). The molecular biology study from Ueki et al. in Japan reveals that the Janus kinase inhibitor tofacitinib suppresses pro-inflammatory cytokine production through suppression of NOD2 expression. The case report from Jensen et al. in the US demonstrates successful treatment of BS with a combination of methotrexate and hydroxychloroquine; two agents thought to interfere with expression and activation of NOD2 and other related immune sensor proteins (15, 16). Although more research is needed, these findings suggest that evaluation and targeting of NOD2 expression levels may be beneficial in shaping an optimal treatment strategy for BS patients.

A more recently described NOD2-associated autoinflammatory disease is Yao syndrome (YAOS, OMIM#617321) (17). YAOS is a systemic inflammatory disease characterized by recurrent fever, dermatitis, arthralgias, distal leg swelling, gastrointestinal, sicca-like symptoms and eyelid swelling to form a complete or partial clinical constellation (18, 19). This disease is linked to multiple *NOD2* variants, such as IVS8 + 158, IVS8 + 158/R702W, IVS8 + 158/L1007fs, IVS8 + 158/V955I (20). These variants are heterozygous in a majority of cases. Originally described as a disease that predominantly affected adult Caucasian women, five articles in this Research Topic expand our understanding of the presentation of YAOS in other age and ethnic groups, as well as refine the associated clinical phenotypes. The large study of 167 Greek patients with autoinflammatory disease by Karamanakos et al. reveals that 7% (12/167) and approximately half of the patients with *NOD2* variants were diagnosed with YAOS and included both pediatric and adult patients. Zhang et al. report on the identification of YAOS in a Chinese population and key differences in disease presentation that include a more balanced sex ratio and a higher proportion of recurrent fever and proteinuria/hematuria than found in Caucasian cohorts. Additionally, similar to genetic analyses of Crohn’s disease in different ethnic groups (20), the *NOD2* variants associated with Chinese YAOS patients were distinct from Caucasian populations. Further substantiating clinical and genotyping features of YAOS, Williamson et al. reports 22 adult patients of European ancestry in the US and Nomani et al. provide a comprehensive study of the clinical phenotype, genotype and therapeutic response of 152 adult YAOS patients. While there is no epidemiologic study of YAOS, the studies in this Research Topic indicate that YAOS is more common than initially thought and impacts a broader spectrum of ethnic backgrounds. We estimate that the prevalence of this disease could approach that of Crohn’s disease (25/100,000), in which up to 10-27% of patients carry *NOD2* variants (21).

These studies also support a new concept in genomic medicine, Genetically Transitional Disease (GTD), in which a gene mutation is necessary, but not sufficient, to cause disease alone. YAOS patients share variants associated with Crohn’s disease (R702W, G908R, L1007fs), but often in combination with other *NOD2* variants or variants associated with other systemic autoinflammatory diseases (SAIDs), such as *MEFV*, *NLRP3*, and *NLRP12* (Nomani et al.) (Karamanakos et al.). GTD underscores the pervasive impact of genetic background together with environment (22). GTD straddles between monogenic and genetic complex/polygenic diseases. This concept applies to many human genetic disorders including certain rheumatic diseases (23). Because genetic testing is crucial for diagnosis of YAOS and differential diagnosis from other SAIDs, an autoinflammatory disease gene panel should be used to include whole NOD2 sequencing to cover intronic variant like IVS8 + 158. Due to the frequent identification of combined *NOD2* variants or *NOD2* with other SAID gene variants in individual patients (Nomani et al.), the “two-hit theory” has been proposed to explain the disease (23) (Nomani et al.). It also suggests that YAOS may also serve as a component of a broader category of autoinflammatory disease termed mixed NLR-associated Autoinflammatory Disease (mixed NLR-AID). Comparisons of mixed NLR-AID could provide insights into common mechanisms of disease, aid in faster diagnosis of these patients, and identify more effective therapeutic interventions for this class of autoinflammatory diseases.

YAOS is a global disease associated with specific *NOD2* variants affecting multiorgan systems and is regarded as a GTD. Patients are frequently encountered in the setting of multidisciplinary care, involving Rheumatology, Allergy, Immunology, Dermatology, Gastroenterology, Infectious Disease, Pulmonology, Cardiology, and Neurology among others. Patients often have recurrent flares and can develop persistent disease with high morbidities and disability, including chronic pain syndrome, gastrointestinal problems and cognitive impairment (19) (Zhang et al.) (Williamson et al.). Future research of the disease using functional assays, population genetics study and bioinformatic analysis would enable to gain deeper understanding of the disease mechanisms and to develop or identify more efficacious drugs.
